# Targeting the Renin-Angiotensin System (RAS) for Neuropsychiatric Disorders

**DOI:** 10.2174/1570159X20666220927093815

**Published:** 2023-01-23

**Authors:** Aline Silva de Miranda, Danielle S. Macedo, Natalia P. Rocha, Antonio L. Teixeira

**Affiliations:** 1 Interdisciplinary Laboratory of Medical Investigation (LIIM), Faculty of Medicine, UFMG, Belo Horizonte, MG, Brazil;; 2 Department of Morphology, Laboratory of Neurobiology, Biological Science Institute, Federal University of Minas Gerais, Belo Horizonte, MG, Brazil;; 3 Department of Physiology and Pharmacology, Neuropharmacology Laboratory, Drug Research, and Development Center, Faculty of Medicine, Federal University of Ceara, Fortaleza, CE, Brazil;; 4 Department of Neurology, The Mitchell Center for Alzheimer’s Disease and Related Brain Disorders, McGovern Medical School, University of Texas Health Science Center at Houston, TX, USA;; 5 Department of Psychiatry and Behavioral Sciences, Neuropsychiatry Program, McGovern Medical School, University of Texas Health Science Center at Houston, TX, USA;; 6 Faculdade Santa Casa BH, Belo Horizonte, Brasil

**Keywords:** Renin-angiotensin system, brain, angiotensin-converting enzyme, neuropsychiatry, Schizophrenia, mood disorder, Alzheimer’s disease, dementia

## Abstract

**Background:**

Neuropsychiatric disorders, such as mood disorders, schizophrenia, and Alzheimer’s disease (AD) and related dementias, are associated to significant morbidity and mortality worldwide. The pathophysiological mechanisms of neuropsychiatric disorders remain to be fully elucidated, which has hampered the development of effective therapies. The Renin Angiotensin System (RAS) is classically viewed as a key regulator of cardiovascular and renal homeostasis. The discovery that RAS components are expressed in the brain pointed out a potential role for this system in central nervous system (CNS) pathologies. The understanding of RAS involvement in the pathogenesis of neuropsychiatric disorders may contribute to identifying novel therapeutic targets.

**Aims:**

We aim to report current experimental and clinical evidence on the role of RAS in physiology and pathophysiology of mood disorders, schizophrenia, AD and related dementias. We also aim to discuss bottlenecks and future perspectives that can foster the development of new related therapeutic strategies.

**Conclusion:**

The available evidence supports positive therapeutic effects for neuropsychiatric disorders with the inhibition/antagonism of the ACE/Ang II/AT1 receptor axis or the activation of the ACE2/Ang-(1-7)/Mas receptor axis. Most of this evidence comes from pre-clinical studies and clinical studies lag much behind, hampering a potential translation into clinical practice.

## 1 INTRODUCTION

Neuropsychiatric disorders, especially severe mental illnesses like mood disorders and schizophrenia, and Alzheimer’s disease (AD) and related dementias, impose a tremendous societal and economic burden, leading to significant morbidity and mortality worldwide [1-5]. It is estimated that major depression and bipolar disorder (BD), the main types of mood disorders, affect approximately 264 million (18.5%) and 45 million (3%) people worldwide, respectively [2, 3]. Both conditions are related with poor quality of life and premature mortality [4]. Increased risk of suicide has been reported in approximately 10 to 20% of patients with BD [6] and in more than 13% of patients with major depressive disorder [7]. Schizophrenia, another chronic disabling psychiatric syndrome, affects approximately 24 million people or 1 in 300 people (0.32%) worldwide [8]. Regarding dementia, an aging-related chronic condition characterized by a progressive cognitive decline [9], it is estimated that around 50 million older adults live with dementia worldwide, and by 2050 this number is projected to triple, with two-thirds of those living in low-income and middle-income countries [5]. AD is the most common cause of dementia, while vascular, frontotemporal, and Lewy body dementias also are important causes [10].

The cellular and molecular mechanisms involved in the pathophysiology of neuropsychiatric disorders are complex and remain to be fully elucidated. A better understanding of their neurobiology is of paramount importance for the development of effective therapies for severe mental illnesses and Alzheimer’s disease and related dementias (ADRD) [9, 11, 12]. Even when considered individually (*e.g*. schizophrenia), neuropsychiatric disorders are heterogeneous and etiologically complex. They encompass a broad spectrum of psychopathological symptoms emerging from diverse pathophysiological mechanisms and are not likely a single disease entity. Probably due to the distinct pathophysiological mechanisms underlying each one of these disorders, many patients do not respond well to the available pharmacological treatments.

Furthermore, some patients do not tolerate the related adverse effects [4, 9, 11-13]. Therefore, identifying novel therapeutic targets and the search for effective treatments for these neuropsychiatric disorders have become goals of the highest priority. Another relevant point for consideration is the evidence showing that people with severe mental illnesses are at increased risk of cardiovascular events, including acute ischemic heart disease and stroke, increasing their morbidity and mortality.

The Renin-Angiotensin System (RAS) comprises a complex and dynamic cascade of peptides, enzymes, and receptors, associated with but not restricted to the cardiovascular system, where it plays pivotal roles in body fluid regulation and homeostasis [14, 15]. Apart from acting as a circulating hormonal system, RAS components are locally expressed in several organs and tissues, including the kidney, brain, and lung, exerting physiological actions through tissue-specific mechanisms [16, 17]. It is now well-accepted that RAS is divided into two main axes: the classical one, including ACE, Angiotensin (Ang) II, Ang type 1 (AT1) receptor (ACE/Ang II/AT1), and the counter-regulatory one, comprising ACE2, Ang-(1-7), Mas receptor (ACE2/Ang-(1-7)/Mas). The classical arm is involved in pro-inflammatory, pro- thrombotic, and pro-fibrotic processes, mainly through the activation of AT1 receptors [18]. Conversely, the counter-regulatory axis plays protective roles by frequently opposing Ang II actions through Mas receptors activation [17, 19]. Accordingly, therapeutic strategies have been designed to inhibit the ACE/Ang II/AT1 axis and stimulate ACE2/Ang-(1-7)/Mas receptor activities [16, 17, 20].

The discovery that RAS components are expressed in several regions of the central nervous system (CNS) opened new opportunities for the investigation of the role of this system in the pathophysiology of a wide range of neuropsychiatric conditions, including neurodegenerative diseases, mood and psychotic disorders [20]. Recently, during the COVID-19 pandemic, as the SARS-CoV-2 uses the RAS component ACE2 as a receptor to invade host epithelial cells and cause organ damage, the discontinuation of ACE inhibitors (ACEIs) and angiotensin II receptor antagonists (ARAs) therapies in older patients with cardiovascular disease and diabetes became a matter of significant debate [21]. Interestingly, these high-risk patients were more likely to develop neuropsychiatric disorders in response to the SARS-CoV-2 infection [22, 23]. Based on the concept that ACEIs and ARAs may stimulate the anti-inflammatory properties of ACE2/Ang-(1-7)/Mas axis, beneficial - not harmful - effects are expected from the treatment with these RAS modulators in the context of COVID-19 and neuropsychiatric disorders [21].

This review aims to discuss emerging evidence from pre-clinical and clinical studies regarding the RAS functions in the CNS and its potential role in the pathophysiology of neuropsychiatric disorders. We also aim to identify bottlenecks and future perspectives for this expanding and promising field that can foster the development of new therapeutic strategies that may reduce the burden of these severe mental illnesses on the individual and society.

## 2 OVERVIEW OF THE RAS

The RAS is classically conceived as a circulating hormonal system that plays a pivotal role in cardiovascular and renal physiology through endocrine, paracrine, and autocrine actions. RAS is composed of several enzymes, inactive and active peptides, primarily implicated in regulating blood pressure and hydroelectrolyte balance [14, 15].

In response to glomerular hypoperfusion, reduced sodium intake, and increased sympathetic nervous system activity, renin, the first enzyme of the RAS, is produced in the juxtaglomerular cells of the afferent renal arteriole and released in the blood stream. Circulating renin hydrolyzes angiotensinogen, the liver-produced protein precursor of the RAS components, in the decapeptide Angiotensin I (Ang I) [14, 24]. This decapeptide has its C-terminal His-Leu residues cleaved by the angiotensin-converting enzyme (ACE), a dipeptidyl-carboxypeptidase, to produce the biologically active octapeptide, Angiotensin II (Ang II).

Ang II is the major active component of the so-called classical RAS and exerts its main effects by binding to a G-protein-coupled receptor known as angiotensin type 1 (AT_1_) [25]. The activation of the classical RAS axis composed of ACE/Ang II/AT_1_ underlies a wide range of pathophysiological events, such as excessive renal sodium reabsorption, abnormal vascular smooth muscle cell contraction, disproportionately high aldosterone secretion, and inappropriate cardiovascular responses [26]. In addition, the AT_1_ receptor also mediates a series of pro-inflammatory, pro-thrombotic, and pro-apoptotic processes associated with several pathological conditions like atherosclerosis, obesity, insulin resistance, and liver diseases [18].

The discovery of the expression of RAS components in different organs and tissues, including the central nervous system (CNS), as well as of other biologically active angiotensin peptides, novel enzymes, and angiotensin receptors, has changed the understanding of the RAS complexity and functions [27]. Particularly relevant for this reconceptualization of the RAS was the characterization of the heptapeptide Ang-(1-7) as a biologically active RAS mediator.

Ang-(1-7) is mainly formed by the activity of the enzyme homolog to ACE, named ACE2, on the C-terminal phenylalanine residue of Ang II. Ang-(1-7) can also be produced directly from Ang I by prolyl-endopeptidase (PEP) and neutral endopeptidase (NEP) or Ang II *via* prolyl-endopeptidase, prolyl-carboxypeptidase. The effects of Ang-(1-7) are mediated by the activation of a G-protein coupled receptor, named Mas receptor, which is expressed, among other tissues, in the brain, heart, kidney, and blood vessels [28, 29]. Through binding on the Mas receptor, Ang-(1-7) often plays a counter-regulatory role by opposing several Ang II/AT_1_ receptor-mediated effects, including vasoconstriction, cell proliferation, inflammation, apoptosis, and tissue fibrosis [17]. Given the opposite roles of Ang II and Ang-(1-7) in different organs and tissues, the concept that the RAS is composed of two major opposite arms has been proposed. Based on this perspective, RAS is envisioned as a dual function system in which vasoconstrictor/proliferative or vasodilator/anti-proliferative effects are driven by the balance between the classical RAS axis formed by ACE/Ang II/AT_1_ and the counter-regulatory axis comprising ACE2/Ang-(1-7)/Mas [17, 26].

In the CNS, the actions of the RAS classical arm were originally associated with cardiovascular and body fluid homeostasis, including, among others, the induction of drinking behavior, salt appetite, vasopressin release, decrease of baroreflex, and enhancement in sympathetic response (Saavedra, 2005). Ang II receptors, like AT_1_, are expressed on neurons of circumventricular regions, *i.e*., brain regions that lack the typical blood-brain barrier, such as the subfornical region located in the roof of the third ventricle, as well as on neurons of the paraventricular nucleus of the hypothalamus and rostral ventrolateral medulla, areas implicated in sympathetic activity regulation [30, 31]. In addition, Ang II receptors were also found in the endothelial cells lining the brain capillaries and microvessels [32]. These findings support that both peripheral and central Ang II may act synergistically to maintain cardiovascular and body fluid homeostasis [30, 33].

Spontaneously hypertensive rats (SHR) present higher expression of AT_1_ in the brain microvessels and middle cerebral artery in comparison with normotensive controls [32]. The activation of the RAS classical axis components in SHR results in cerebral vasoconstriction and decreased vascular capacity to dilate during hypoperfusion, underlying the SHR vulnerability to cerebral ischemia [34, 35]. Conversely, acute intravenous or prolonged subcutaneous administration of candesartan, a selective antagonist of AT_1_, normalized the capacity of cerebral arteries to dilate, improved arterial compliance and collateral flow, and reduced the size of brain infarct and tissue swelling following middle cerebral artery occlusion in SHR [35, 36]. The protective effects of candesartan in SRH following ischemia were also associated with attenuation of brain oxidative stress and inflammation [32, 37]. Importantly, a growing body of clinical studies showed that ARAs, like losartan and eprosartan, were more effective in reducing stroke incidence compared with anti-hypertensive drugs from other classes, such as atenolol and nifedipine [38, 39]. In addition, evidence provided by the TROPHY trial suggests that the ARA’s beneficial effects on stroke may last even after the drug administration has ceased [40, 41]. Taken together, these studies support the role of the ACE/Ang II/AT_1_axis in the pathophysiology of cerebrovascular conditions, indicating that the inhibition and/or antagonism of RAS classical components like AT_1_ may be of therapeutic value in CNS dysfunction.

With the discovery of components of the to be called counter-regulatory RAS axis (formed by ACE2, Ang-(1-7), and the Mas receptor), studies were conducted to understand whether the Ang-(1-7) heptapeptide had any of the Ang II effects. An *in vitro* study revealed that Ang-(1-7) was as potent as Ang II in inducing vasopressin release from the rat hypothalamo-neurohypophysial system. This finding provided evidence that Ang-(1-7) is not only endogenously generated but is also a centrally active peptide [42]. Immunocytochemical analysis in the rat forebrain demonstrated that Ang-(1-7) is expressed in key areas associated with the regulation of endocrine functions, including the paraventricular, supraoptic, and suprachiasmatic nuclei, median eminence, and neurohypophysis, reinforcing the idea that Ang-(1-7) is an important component of vasopressin release control [43]. In opposition to Ang II effects, intracerebroventricular infusion of Ang-(1-7) improved baroreflex control of heart rate in normotensive rats and SHR [44, 45]. Similar findings were reported following Ang-(1-7) injections in the rat nucleus tractus solitarii [46]. ACE2 gene transfer into the nucleus tract solitarii of SHR, resulting in long-term overexpression of ACE2, also improved baroreceptor heart rate reflex due to RAS counter-regulatory axis activation [47]. Moreover, intracerebroventricular infusions of Ang-(1-7) for 4 weeks reduced the expression of Ang II and AT_1_, and induced anti-oxidative and anti-apoptotic effects in the brain of SHR without changing blood pressure [48]. In line with these findings, intracerebroventricular infusion of Ang-(1-7) over 6 weeks in stroke-prone SHR, an established animal model of hypertension-induced hemorrhagic stroke, increased survival time and improved neurological status [49]. Altogether, these studies indicate that the RAS counter-regulatory axis components might be potential therapeutic targets to prevent hypertension-related cerebrovascular diseases. Despite this convincing evidence on the beneficial role of the RAS counter-regulatory axis in experimental models of stroke, evidence from clinical studies involving patients with cerebrovascular diseases is still missing. More studies are also warranted to define whether the administration of ACE2 activators and/or Mas receptor agonists may exert protective effects in cerebrovascular disorders.

## 3 THE RAS BEYOND VASCULAR MODULATION: FOCUS ON STRESS RESPONSE

The description of RAS components in key brain areas belonging to the hypothalamic-pituitary-adrenal axis (HPA), like the hypothalamic paraventricular nucleus and the pituitary [50], prompted the investigation of the potential role of this system in neuropsychiatry disorders through neuroendocrine modulation [51-54].

Acute and chronic stress can increase Ang II circulating and brain tissue levels, and enhance the expression of AT_1_ in the parvocellular portion of the paraventricular nucleus, the site of corticotrophin-releasing hormone (CRH) formation. Increased CRH production, in turn, contributes to HPA axis hyperactivation, which results in increased pituitary adrenocorticotropic hormone (ACTH) release and secretion of glucocorticoid steroid hormones from the adrenal cortex [50, 55, 56]. The adrenal corticosterone release in response to HPA axis hyperactivation and/or dysfunction may exert neuronal-toxic effects, contribute to neurotransmission imbalance and neurodegeneration, all implicated in neuropsychiatric disorders [51, 53, 54]. Of relevance, the inappropriate response to stress is a well-established pathogenic factor for neuropsychiatric conditions [54]. Ang II and AT_1_ are also expressed in other brain areas implicated in stress-related and neuropsychiatric disorders, such as the amygdala, hippocampus, and prefrontal cortex, suggesting the involvement of RAS in their pathophysiology [57]. Furthermore, Ang II regulates vasopressin and oxytocin release by the hypothalamus [58], neurohormones related to the regulation of stress response [59] and complex social behaviors, such as aggressive behavior. Orally active vasopressin V1a receptor antagonists are in clinical trials for the treatment of autism spectrum disorder [60].

Experimental and clinical studies investigating the effect of AT_1_ receptor antagonists (ARA) and ACE inhibitors in the regulation of stress response have also reinforced the potential role of RAS in stress-related disorders [61-63]. For instance, peripheral administration of candesartan, an ARA, or ramipril, an ACE inhibitor, for 5 weeks attenuated the stress sensitivity of the HPA axis in SHR, independently of their capacity to lower blood pressure [64]. Additionally, acute (single dose) or chronic (2 weeks) systemic administration of the ARA losartan enhanced the consolidation of fear extinction without affecting fear acquisition, baseline anxiety, blood pressure, and neuroendocrine stress measures in a murine model of posttraumatic stress disorder (PTSD) [63]. In line with these findings, ARAs and/or ACE inhibitors displayed protective effects among individuals presenting PTSD-related symptoms. Conversely, other anti-hypertensives, including beta-blockers, calcium channel blockers, and diuretics, were not significantly associated with PTSD symptom reduction [62]. Taken together, these studies suggest that RAS may be a promising therapeutic target to improve resistance to stress and neuropsychiatric conditions related to HPA axis dysfunctions, including anxiety and mood disorders.

To a lesser extent, the involvement of the RAS counter-regulatory arm in stress-related disorders has also been reported. In contrast to the RAS classical arm, the ACE2/Ang-(1-7)/Mas receptor axis displays protective effects in response to stress [65-69]. Intravenous or intracerebroventricular administration of Ang-(1-7) or of an ACE2 activator (XNT) in Wistar rats abolished the tachycardia induced by air-jet stress or treatment with the beta-adrenergic agonist isoproterenol [65]. Injection of Ang-(1-7) in the basolateral amygdala, a key brain region involved in physiological responses to emotional stress, attenuated tachycardia, and blood pressure elevation in rats. These positive effects were prevented by the Mas receptor antagonist, A-779, suggesting that Ang-(1-7) modulates the cardiovascular response to emotional stress by activating the Mas receptors in the basolateral amygdala [67]. Similar findings were found in a study with transgenic rats overexpressing Ang-(1-7) (TGR(A1-7)3292) [69]. One week of intracerebroventricular infusions of Ang-(1-7) prevented gastric ulcers induced by 2 hours of cold-restraint stress in rats. This protective effect was associated with a reduction in the levels of stress-related hormones, including Ang II and glucocorticoid, in the blood and stress-related brain regions, such as the hypothalamus, hippocampus, prefrontal cortex, and amygdala [66]. Finally, genetic deletion of the Mas receptor in mice increased contextual fear memory and slowed its extinction [68]. As fear memory extinction impairment can be interpreted as a maladaptive behavior like the one seen in PTSD, these authors proposed the Mas-KO mice as an animal model of PTSD [68]. Although mainly pre-clinical, the evidence provided by these studies sheds light on the role of the RAS counter-regulatory arm in modulating the response to stress and its therapeutic potential for stress-related conditions, including neuropsychiatric disorders.

The RAS seems to participate in other processes known to be involved in the neurobiological basis of neuropsychiatric disorders, such as oxidative stress and inflammation [20, 70, 71]. Sprague-Dawley rats repeatedly exposed to systemic injections of lipopolysaccharide (LPS), a well-known immune system activator, displayed depressive and anxiety-like behaviors alongside microglial activation and increased hippocampal expression of the pro-inflammatory cytokines IL-1β and IL-6, and the nitric oxide (NO)-producing isoenzyme inducible NO synthase (iNOS) and cyclooxygenase 2 (COX-2). Oral administration of the ARA candesartan over 2 weeks caused anxiolytic and antidepressant effects, and attenuated brain inflammation and oxidative stress [72]. Similar anti-inflammatory findings were reported in animal models of epilepsy, brain ischemia, traumatic brain injury, and neurodegeneration [73-77] as well as in patients with inflammatory diseases [77-79]. Pre-clinical and clinical studies also support an anti-inflammatory and antioxidant role for the RAS counter-regulatory axis [17, 20]. In this scenario, the potential of RAS modulators like ACE inhibitors, ARAs, ACE2 activators, and/or Mas receptor agonists as therapeutic strategies for neuropsychiatric disorders is a matter of growing interest.

## 4 RAS AND MOOD DISORDERS

Mood disorders, previously known as affective disorders, are a complex group of chronic, recurrent psychiatric illnesses characterized by a constellation of emotional, cognitive, behavioral, and somatic symptoms. They are associated with significant disability, being a leading cause of morbidity and mortality worldwide [2]. The interplay between genetic and environmental/stress factors has been implicated in the pathogenesis of these conditions. The two main instances of mood disorders are major depressive disorder (MDD), also known as unipolar depression, and bipolar disorder (BD) [80, 81]. The currently available pharmacological therapies are often ineffective (~ 30%) and/or poorly tolerated due to adverse effects [4, 13, 82]. Therefore, the search for new treatment approaches for mood disorders has become a high priority to reduce the burden of these illnesses.

Clinical and experimental studies have shown the involvement of RAS components in depressive disorders [83-89]. Initial evidence came from studies conducted in the 1980s with hypertensive patients with depression that revealed positive effects of the ACE inhibitor captopril on both blood pressure control and depressive symptoms [90-94]. In addition, the antidepressant effects of captopril were associated with reduced circulating levels of cortisol [94]. Corroborating these preliminary studies, captopril and enalapril treatments ameliorated depressive and anxiety symptoms in hypertensive patients compared with hypertensive individuals taking other anti-hypertensive drugs [83]. Hypertensive patients under RAS modifying therapy with either ACE inhibitors or ARAs presented lower rates of antidepressant use compared with patients using other anti-hypertensive drugs [85]. Lower frequency of anti-depressants use was also reported among patients with type 1 diabetes and diabetic nephropathy taking RAS-active agents compared with diabetic patients without RAS-modifying treatment [86]. Moreover, the ARA candesartan administered for at least 3 months attenuated HPA axis hyperactivation, as shown by decreased levels of circulating cortisol, and improved depressive symptoms in patients with diabetes mellitus type 2 [95]. Although there are no randomized controlled trials (RCTs) investigating the potential anti-depressant effects of RAS modifying drugs in humans, these observational or case-control studies support RAS modulators as a promising therapeutic strategy in MDD. Interestingly, a recent pharmacogenomic survey to identify candidate drugs for repositioning in treatment-resistant depression indicated telmisartan and nelivaptan among the potential therapeutic compounds [12].

Polymorphism studies also support a role for RAS classical arm components in MDD [52, 96, 97]. Variants of the ACE gene, rs4291 and the D allele, related to higher ACE serum activity and HPA axis hyperactivity, have been associated with mood disorders [52, 96]. A polymorphism study conducted with DNA extracted from buccal cells of depressed patients found a significant association between the AT1 receptor (A1166C) CC genotype and depressive symptoms, supporting the hypothesis that increased activity of the RAS classical axis may increase the risk for depression [97].

The involvement of the RAS classical arm in the pathophysiology of depressive disorders has also been reported in pre-clinical studies. Captopril was as efficient as the anti-depressants imipramine and mianserin in inducing antidepressant-like effects in mice subjected to the forced swim test [84, 87-89, 98]. Similar findings were observed in rats on the learned helplessness test [98]. Regarding the anti-depressant properties of ARAs, systemic administration of telmisartan attenuated diabetes-induced depression in rats, as indicated by increased immobility in the forced swim test. Telmisartan improved depressive-like behavior in parallel with reducing serum cortisol levels, nitric oxide, and pro-inflammatory cytokines IL-1β and IL-6 [88]. In the unpredictable chronic mild stress paradigm, a more ecological model of depressive-like behavior in rodents, the ARAs irbesartan and valsartan showed antidepressant-like activity. This effect was associated with reduced oxidative stress and elevated brain levels of serotonin (5-hydroxytryptamine, 5-HT), as well as restored hippocampal neurogenesis and expression of BDNF [87, 89]. Transgenic mice lacking angiotensinogen presented less depressive-like behavior compared with wild-type animals, but no changes were found in anxiety-like behavior or cognition [99]. In these transgenic mice, angiotensinogen function is completely abolished [99]. The absence of angiotensinogen hampers the production of Ang II (20), which explains, at least in part, the attenuation of the depressive-like behaviors reported in these animals.

The counter-regulatory RAS arm seems to have a protective role in mood disorders, but the evidence is still scarce and based mainly on pre-clinical studies. Transgenic rats with a down-regulated synthesis of angiotensinogen in brain glial cells (TGR(ASrAOGEN) 680) displayed depressive-like behavior in the forced swim test along with decreased levels of 5-HT and its metabolite 5-hydroxyindoleacetic acid (5-HIAA) in the hippocampus, frontal and parietal cortices [100, 101]. Short-term (7 days) intracerebroventricular injection of Ang-(1-7) was able to reverse depression-like phenotype in these genetically modified rats with low brain angiotensinogen [101]. The same protocol of intracerebroventricular infusion of Ang-(1-7) was able to prevent depressive-like behavior in the forced swim test in transgenic rats with hypertension due to an additional renin gene (TGR(mRen2)27), reinforcing the protective effect against depression of the RAS counter-regulatory axis [102].

Studies investigating the relationship between RAS and BD are even more limited. The challenges of investigating RAS-related dysfunctions and their involvement in the pathophysiology of BD rely, at least in part, on the cyclic nature of this condition, *i.e*. the fluctuation between cycles of depression and mania. A single polymorphism study conducted with BD patients found a strong association between an angiotensinogen M235T genotype and increased susceptibility to BD [103]. A more recent cross-sectional study investigated the levels of RAS-related molecules in the plasma of BD patients and healthy controls. BD patients experiencing mood episodes had increased plasma levels of ACE compared with controls, but no associations were found between the levels of RAS components and depressive or manic symptoms [104]. In an experimental model of mania induced by amphetamine in mice, the oral administration of the ARA candesartan prevented manic-like behavior. The antimanic-like effect was associated with changes in brain antioxidant, anti-inflammatory, and neurotrophic pathways [105]. Given the antidepressant effect of RAS modulators, their associated anti-manic potential might suggest a broader application of these drugs for mood regulation.

## 5 RAS AND SCHIZOPHRENIA

Schizophrenia usually affects young individuals just as they gain independence, and it can leave them disabled for the rest of their lives. This severe mental illness is one of the worst conditions afflicting humankind in terms of personal and economic costs. Despite advances in the treatment of schizophrenia, only positive symptoms, *i.e*. delusions and hallucinations, are controlled with antipsychotic drugs. At the same time, negative (reduced expression of emotions, poverty of speech, anhedonia, asociality) and cognitive (difficulty concentrating, organizing, planning, and problem solving) symptoms are not efficiently handled by these medications.

Like other severe mental illnesses, schizophrenia has a complex, heterogeneous, and polygenic genetic architecture, with multiple common genetic variants having only minor individual effects (*e.g*., single nucleotide polymorphisms) and rare highly penetrant genetic variants having bigger impacts (*e.g*., copy number variations) [106]. In a Han Chinese population, an ACE gene insertion/deletion polymorphism was associated with schizophrenia risk and severity of depressive symptoms [107]. In a Spanish population, the D allele of the ACE gene was a protective factor lowering the risk of developing schizophrenia by 50% [108].

A higher ACE activity was found in the plasma of chronic schizophrenia patients, being positively correlated with plasma levels of the pro-inflammatory cytokines interferon gam (IFN-γ) and interleukin (IL)-17A, and associated with cognitive deficits [109, 110]. A study evaluating ACE activity in first-episode patients revealed that these patients showed increased ACE activity, mainly in the ACE DD genotype subgroup, but without association with symptom improvement after antipsychotic treatment [111]. Regarding the ACE protein levels, schizophrenia patients had decreased levels compared with controls, without alterations in ACE2, Ang-(1-7) and Ang II levels [112]. In line with these findings, a recent study involving 40,675 patients with schizophrenia and 64,643 controls, 20,352 patients with BD and 31,358 controls, and 135,458 patients with MDD and 344,901 controls revealed that a 1-SD lower blood expression of the ACE gene was associated with a lower systolic blood pressure of 4.0 (95% CI,2.7-5.3) mm Hg, but increased risk of schizophrenia (odds ratio (OR), 1.75; 95% CI) [113]. Notwithstanding, another recent study showed weak evidence for higher systolic blood pressure and increased schizophrenia risk [114]. The liability to schizophrenia increases the risk of heart problems, therefore the monitoring and treatment of cardiovascular health should be implemented in the early stages of psychosis aiming to decrease the related mortality [114]. In fact, people with schizophrenia have a shorter life expectancy than the general population, at least in part due to a higher risk of cardiovascular diseases. Accordingly, RAS alterations are related to increased schizophrenia risk and possibly cardiovascular risk in these patients.

One of the core symptoms of schizophrenia is cognitive impairment. Following the evidence from preclinical models of neurodegenerative diseases that candesartan has direct neuroprotective properties [115], a study conducted by our group revealed that low dose of candesartan administered during peri-adolescence prevented the development of schizophrenia-like symptoms and hippocampal pro-inflammatory alterations in male and female mice submitted to the two-hit model of schizophrenia [116]. This drug also seemed to be effective against haloperidol-induced tardive dyskinesia in rats [117]. Pro-inflammatory changes are proposed as underlying mechanisms of cognitive impairment [118] and tardive dyskinesia [117], and both can be modulated by the RAS [20]. Furthermore, ARAs inhibit kynurenine aminotransferase II (KAT II) in the brain [119], and this effect can lead to decreased levels of kynurenic acid (KYNA), an endogenous antagonist of NMDA receptors whose levels are increased specifically in the CNS of patients with schizophrenia [120].

## 6 RAS IN ALZHEIMER’S DISEASE AND RELATED DEMENTIA

Alzheimer's disease (AD) and related dementias (ADRD) are the most common neurodegenerative diseases and the main causes of dementia worldwide [121, 122]. Aging is the main risk factor for ADRD, whose incidence and prevalence are expected to increase in the next few years due to longer life expectancy. ADRD are devastating neurodegenerative dementias for which there are no effective treatments. Strategies aiming at preventing or slowing disease progression are therefore urgently needed [122].

The major neuropathological changes of patients with ADRD are neuronal death, synaptic alterations, brain inflammation, and the presence of protein aggregates. The protein aggregates appear in the form of extracellular amyloid plaques and intra-neuronal neurofibrillary tangles composed of amyloid-beta (Aβ) peptide and hyperphosphorylated Tau, respectively [123]. Of note, the accumulation of misfolded protein aggregates is selectively (but not exclusively) associated with discrete neurodegenerative diseases. For example, α-synuclein deposits are commonly observed in dementia with Lewy bodies, huntingtin in Huntington’s disease, and TAR DNA-binding protein in frontotemporal dementia. However, there is a substantial overlap of pathological abnormalities in neurodegenerative dementias, leading to mixed pathologies, *i.e*. multiple protein aggregates in the same brain [124-128]. The processes of protein misfolding and oligomerization begin years or decades before the onset of clinical symptoms and substantial brain damage [129, 130].

Although protein misfolding and aggregation are regarded as the main contributors to neurodegeneration in ADRD, additional mechanisms are involved in their development and/or progression. Currently, the complexity and multicausality of ADRD are well-recognized, with research showing a plethora of mechanisms related to their pathophysiology, including inflammation and oxidative stress [5, 131]. As our understanding of the RAS has evolved, including the recognition that all RAS components are present in the brain [14, 132, 133], changes in the RAS have been linked to the pathophysiology of ADRD [20].

Preclinical studies have shown an inverse correlation between Ang-(1-7) levels and tau hyperphosphorylation in brain regions directly involved in ADRD pathophysiology (*i.e*., cerebral cortex and hippocampus) of both senescence-accelerated mouse prone 8 (SAMP8) mice (an animal model of sporadic AD) and P301S mice (an animal model of tauopathy). These results suggest that the RAS is involved in ADRD pathophysiology, probably *via* modulation of tau hyperphosphorylation [134].

There is also evidence of RAS imbalance in patients with AD. A post-mortem study reported decreased activity of ACE2 in cortex samples of patients with AD, which correlated negatively with Aβ and phosphorylated tau pathology. Noteworthy, the Ang II/Ang (1-7) ratio was reduced in the AD brains compared with age-matched controls [135]. In line with these findings, one study found that a decrease in plasma levels of Ang-(1-7) correlated with cognitive decline in AD patients, suggesting Ang-(1-7) as a potential biomarker for AD [136]. A recent study has shown that not only the plasma levels of Ang-(1-7) are decreased in patients with AD in comparison with controls, but they correlate with white matter hyperintensities lesion volumes, pointing out a possible interaction between Ang-(1-7) and cerebrovascular lesions in AD [137]. Finally, cerebrospinal fluid levels of ACE are decreased in patients with AD in comparison with age-matched controls and correlated with reduced Aβ_42_ levels, a well-established marker of Aβ amyloid accumulation in the brain [19].

Because the RAS has been consistently linked to neurodegeneration, studies have assessed potential treatments for ADRD focusing on either preventing the harmful effects of Ang II through AT_1_ receptors or the neuroprotective role of the ACE2/Ang-(1-7)/Mas axis activation. Classical RAS-modifying drugs, *i.e*., ACE inhibitors and ARAs, have been evaluated in preclinical and clinical studies targeting ADRD. Telmisartan reduced amyloidopathy after transient middle cerebral artery occlusion in stroke-resistant SHR rats [138]. In addition, treatment with ARAs prevented and/or attenuated stress-induced cognitive impairment in rodents [139-142].

Observational studies focusing on the association between the use of drugs targeting the RAS and ADRD risk confirmed the aforementioned experimental data. A prospective study on RAS-modifiers, mainly ARAs, showed their potential in reducing the incidence and progression of ADRD [143]. In addition, a 6-month treatment of hypertension with the ARA telmisartan in patients with AD resulted in beneficial effects on cognition and cerebral blood flow compared to anti-hypertensive drugs with different mechanisms of action, such as the calcium channel blocker amlodipine [144]. A meta-analysis found that the use of drugs interacting with the RAS (both ACE inhibitors and ARAs) was significantly associated with a reduced risk of AD and aging-associated cognitive impairment [145]. It is worth emphasizing that ARAs are safe and clinically effective against major risk factors for ADRD, including hypertension, vascular disease, chronic kidney disease, diabetes, and metabolic syndrome. Altogether, there is enough evidence to consider the development of studies assessing the potential of ARAs in preventing/treating ADRD [70].

The effects of classical RAS inhibitors (especially ARAs) are, at least in part, related to the activation of the counter-regulatory RAS axis. Accordingly, the stimulation of the ACE2/Ang-(1-7)/Mas receptor axis might be *per se* a promising therapeutic target for ADRD. Evidence from preclinical studies corroborated this hypothesis. The intracerebroventricular infusion of Ang-(1-7) prevented or ameliorated cognitive impairment and AD-related pathology in 5XFAD mice [146] and AD-like rat model resulting from streptozotocin-induced diabetes [147]. The protective effects of Ang-(1-7) were inhibited by the co-administration of A-779 (a Mas receptor antagonist), indicating that Ang-(1-7) neuroprotection was mediated by the Mas receptor [147]. Ang-(1-7) also attenuated behavioral and pathological changes observed in a rat model of AD induced by the intracerebroventricular injection of Aβ_42_, an effect inhibited by the Mas receptor antagonist A-779 [148]. In line with these findings, AVE0991 (an analogue of Ang-(1-7)) ameliorated spatial cognitive impairment, inhibited neuroinflammation, and lessened neuronal and synaptic damage in APP/PS1 mice [149]. Moreover, the intraperitoneal injection of diminazene aceturate (DIZE), an established activator of ACE2, decreased Aβ levels in the hippocampus and restored cognitive impairment in Tg2576 mice [150].

## 7 CHALLENGES AND OPPORTUNITIES

**Fig. (1):**
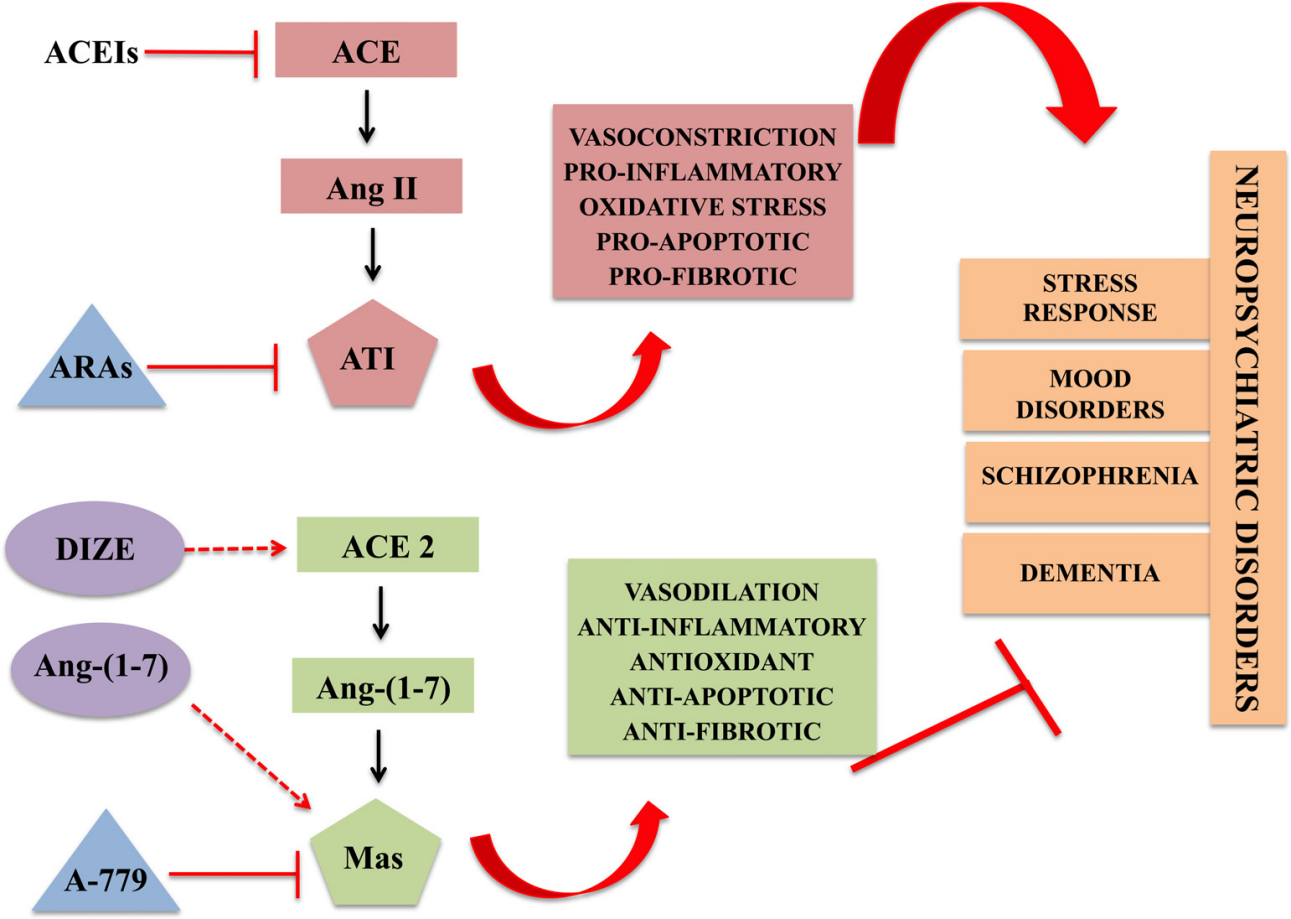
Potential role of Renin-Angiotensin System (RAS) components in the development and progression of neuropsychiatric disorders. The classical RAS axis, composed by ACE/Ang II/AT_1_ receptors, can induce inflammation, oxidative stress and apoptosis, processes implicated in the pathophysiology of neuropsychiatric disorders. The counter-regulatory axis, which encompasses ACE2/Ang-(1-7)/Mas receptors, counterbalances these mechanisms, and seems to exert neuroprotective effects. In this context, drugs capable of inhibiting/antagonizing the classical RAS axis, like ARBs and ACEIs, or stimulating the RAS counter-regulatory axis, such as the ACE 2 activator diminazene aceturate (DIZE) and the Mas receptor agonists including Ang-(1-7), are promising therapeutic strategies for neuropsychiatric disorders. **Abbreviations**: Angiotensin converting enzyme (ACE); Angiotensin converting enzyme-2 (ACE 2); Angiotensin II (Ang II); Angiotensin-(1-7) (Ang-(1-7)); Angiotensin II receptor type 1 (AT_1_); ACE inhibitors (ACEIs); Angiotensin receptor antagonists (ARAs); ACE 2 activator diminazene aceturate (DIZE).

The recognition that RAS components are involved in the HPA axis activity, oxidative stress and inflammation, all implicated in the pathophysiology of neuropsychiatric disorders [57], has stimulated the investigation of RAS modulators as potential therapeutic candidates for these conditions (Fig. **1**). The established safety and efficacy of ACEIs and ARAs for cardiovascular and metabolic diseases, such as hypertension, heart failure, and diabetes [151], suggest the potential scalability of these strategies and their use in patients with neuropsychiatric disorders who frequently have these medical comorbidities. Besides symptomatic improvement, as cerebrovascular conditions are high-risk factors for the development of neurodegenerative and mood disorders [152-154] and given the efficacy of RAS modulators in their control, these drugs may contribute to preventing the occurrence of these neuropsychiatric disorders. In the context of prevention, patients in the prodromal and/or early phases of schizophrenia might also benefit from ARAs based not on vascular risk factors but on the preclinical evidence showing their effects on inflammatory and glutamatergic pathways.

Despite this promising scenario, it is important to acknowledge that the available data supporting the role of RAS in neuropsychiatric disorders as well as its therapeutic potential is mostly pre-clinical. Even these experimental or pre-clinical studies are still largely descriptive and lack mechanistic insights that directly link RAS to the pathophysiology of neuropsychiatric disorders. The complex neurobiological basis of these disorders makes the development of appropriate animal models particularly challenging, which hampers, at least in part, the investigation of the underlying pathophysiological mechanisms. For instance, there are no available animal models of BD able to cover the full spectrum of the disease by mimicking mood/emotional state changes across manic and depressive poles. The limited understanding of the fundamental cellular and molecular mechanisms underlying these neuropsychiatric disorders is also a major constraint to developing effective therapies for these conditions.

Another important gap to be considered is the lack of clinical trials aiming to investigate the effects of RAS modulators in neuropsychiatric disorders. For example, a pharmacogenomic study proposed the ARAs telmisartan and nelivaptan as potential candidate drugs for treatment-resistant depression [12], but no clinical trials have been carried out so far. For some conditions like BD, even descriptive studies of RAS components in humans are scant. Our research group conducted one of the very few studies in BD reporting decreased ACE levels among individuals with BD compared to controls; however, no correlations were found between ACE levels and depressive or manic symptoms [104].

A growing body of evidence has confirmed the opposing actions of Ang-(1-7) in relation to Ang II in different biological processes (*e.g*., apoptosis, angiogenesis, inflammation) and that the activation of the ACE2/Ang-(1-7)/Mas receptor axis might exert positive and/or protective effects in models of and patients with neuropsychiatric disorders [19]. Nevertheless, this non-canonical axis remains understudied in this context. More specifically, studies to investigate whether ACE2 activators and/or Mas receptor agonists might benefit patients with neuropsychiatric disorders are highly warranted. A major drawback to therapies focused on improving brain dysfunctions is delivering a drug that must cross the blood-brain barrier, reaching its target without intracerebrovascular injection or other complex methods. Another major concern regarding the delivery of short peptides like Ang-(1-7) is protecting against proteolytic degradation, which contributes to a short plasma half-life. Some solutions have been proposed, like the encapsulation of Ang-(1-7) in cyclodextrin or microencapsulated in biodegradable polymers or liposomes. These strategies may enable the administration of Ang-(1-7) as an oral formulation, which might allow the treatment of a broad range of patients [155].

The potential role of angiotensin II type 2 receptors (AT2) in the brain also remains to be fully appreciated. Despite the fact that Ang II effects are mainly mediated by AT1 receptors, Ang II can also bind to AT2 receptors whose signaling often opposes the effects of the AT1 receptor [156]. Activation of AT2 receptors seems to prevent cognitive impairment in animal models of cerebrovascular diseases, AD and vascular dementia [157-161]. Therefore, although the ACE2/Ang-(1-7)/Mas receptor axis has been highlighted as the RAS main protective arm, activation of AT2 receptors might also exert beneficial or neuroprotective effects [162, 163]. Further studies are necessary to better address the involvement of AT2 receptor in psychiatric disorders and neurodegenerative diseases as well as its potential as a therapeutic target.

## CONCLUSION

The available evidence supports a role for RAS in the pathophysiology of neuropsychiatric disorders as well as the use of RAS modulators as potential therapies. However, most of this evidence comes from pre-clinical studies and clinical studies lag much behind hampering a potential translation into clinical practice.

## LIST OF ABBREVIATIONS

AD: Alzheimer’s Disease
BD: Bipolar Disorder
ADRD: Alzheimer’s Disease and Related Dementias
RAS: Renin-angiotensin System
CNS: Central Nervous System
ACE: Angiotensin-Converting Enzyme
PEP: Prolyl-Endopeptidase
NEP: Neutral Endopeptidase
SHR: Spontaneously Hypertensive Rats
HPA: Hypothalamic-Pituitary-Adrenal Axis
CRH: Corticotrophin-Releasing Hormone
PTSD: Posttraumatic Stress Disorder
LPS: Lipopolysaccharide
